# Balancing Endoscopy and Surgery in Choledocholithiasis: A 12-Year Real-World Analysis From a Brazilian Tertiary University Hospital

**DOI:** 10.7759/cureus.92083

**Published:** 2025-09-11

**Authors:** Otávio B Ceribeli, Philipe F Tafner, Martinho A Gestic, Murillo P Utrini, Francisco Callejas-Neto, Everton Cazzo

**Affiliations:** 1 Surgery, State University of Campinas (UNICAMP), Campinas, BRA

**Keywords:** biliary tract surgical procedures, cholangitis, choledocholithiasis, endoscopic retrograde cholangiopancreatography (ercp), treatment outcome

## Abstract

Background

Choledocholithiasis is a common biliary disorder, with endoscopic retrograde cholangiopancreatography (ERCP) as the preferred therapy. However, anatomical complexity, prior surgery, or severe cholangitis often require surgical intervention. Data from the Latin American tertiary centers remain scarce.

Objective

To evaluate the real-world management of choledocholithiasis in a Brazilian university hospital over 12 years, comparing outcomes between ERCP and surgical approaches.

Methods

This was a retrospective, single-center study including a convenience sample of 251 consecutive patients treated between 2011 and 2023. Patients were stratified into exclusive ERCP, upfront surgery, or surgery following ERCP failure. Demographic, clinical, laboratory, and procedural variables were analyzed, focusing on complications, length of stay, and mortality.

Results

ERCP was performed exclusively in 77.8% of patients, while 22.2% required surgery, half after failed ERCP. Surgical patients had higher rates of previous cholangitis (46.4% and 42.9% vs. 26.7%, *p*=0.04) and prior abdominal surgery. Laboratory differences included lower international normalized ratio (INR) in upfront surgery (*p*<0.001) and higher aspartate aminotransferase and alanine aminotransferase (AST/ALT) in ERCP patients (*p*=0.003 and *p*=0.002). ERCP-related complications occurred in 9% of procedures, while surgical groups showed higher complication rates (32.1% upfront, 25% after ERCP vs. 12.3% ERCP-only, *p*=0.01). Hospitalization was significantly longer in surgical patients (9 ± 4.5 and 12 ± 12.3 days vs. 4.7 ± 5.7 days, *p*=0.0002), whereas ICU stay did not differ (*p*=0.46). In-hospital mortality rates were 5.1% (ERCP), 7.1% (upfront surgery), and 8.3% (surgery after ERCP), with no significant difference between groups (*p*=0.85).

Conclusions

Our findings represent institutional real-world experience, highlighting ERCP as the first-line therapy while surgery remains essential in complex or refractory cases. Conclusions are limited by the retrospective design and potential selection bias. Strengthened referral pathways and early risk stratification are essential to optimize outcomes in public healthcare systems.

## Introduction

Cholelithiasis is a common condition, affecting approximately 20 million individuals in the United States and accounting, together with cholecystitis, for nearly 1.4 million emergency department visits annually. Among patients undergoing cholecystectomy, 10-15% present with choledocholithiasis, with incidence increasing with age [1-3].

Common bile duct (CBD) stones are classified as primary or secondary. Primary stones, which are less frequent, are usually associated with bile stasis in conditions such as cystic fibrosis, bile duct dilation, or the presence of diverticula, and may develop in intra- or extrahepatic ducts. Secondary stones, more prevalent in Western countries, result from the migration of gallbladder stones into the CBD [1,2].

Diagnosis combines laboratory evaluation with imaging methods. Abdominal ultrasound is the most common initial exam, while magnetic resonance cholangiopancreatography (MRCP) and endoscopic ultrasound increase diagnostic accuracy. Endoscopic retrograde cholangiopancreatography (ERCP) is now reserved mainly for therapeutic purposes, once high probability or confirmation of CBD stones is established [1,2,4].

Risk stratification considers patients with a visible stone on ultrasound, acute cholangitis, or bilirubin >4 mg/dL associated with CBD dilation (>6 mm, or >8 mm after cholecystectomy) as high risk. Intermediate-risk patients include those with abnormal liver function tests, age >55 years, or CBD dilation without direct evidence of stones. Patients without these features are classified as low risk [1].

ERCP is the treatment of choice in most cases, usually followed by laparoscopic cholecystectomy. In selected cases, laparoscopic cholecystectomy with bile duct exploration may be an alternative. However, 10-15% of stones cannot be removed endoscopically, requiring surgical intervention. Factors that hinder endoscopic management include large (>1.5 cm), multiple, atypical, intrahepatic stones, or altered anatomy of the biliary tract [2]. In recent years, combined approaches such as laparo-endoscopic ‘rendezvous’ techniques and advanced laparoscopic bile duct exploration have emerged as alternatives that may improve stone clearance rates in selected cases, although their adoption remains limited in many public healthcare systems due to resource and expertise constraints [5,6].

Although ERCP is considered the gold standard, its availability is heterogeneous across different regions, directly influencing therapeutic decisions. In this scenario, laparoscopic bile duct exploration (transcystic or transductal) and open surgical approaches (such as biliodigestive diversion or transduodenal sphincteroplasty) remain valid options in selected cases [2,7].

Given the variability in access to ERCP and the lack of consensus in the literature regarding the relative use of endoscopic versus surgical strategies, the objective of this study was to evaluate the real-world management of choledocholithiasis in a tertiary Brazilian university hospital over a 12-year period, comparing outcomes between ERCP and surgical approaches.

## Materials and methods

Study design and setting

This was a retrospective, longitudinal, observational study based on a convenience sample of all consecutive patients meeting the inclusion criteria between 2011 and 2023. It was based on the review of medical records of patients who underwent ERCP or surgical procedures (laparoscopic or open bile duct exploration) at the Clinical Hospital of the University of Campinas (HC-Unicamp) between 2011 and 2023. The study was approved by the State University of Campinas Research Ethics Committee (approval no. 6.552.262). Written informed consent was provided by all patients or, when applicable, by their legal representatives.

Study population

Patients were identified through the institutional electronic medical record system. Inclusion criteria were: (1) age ≥18 years; (2) diagnosis of choledocholithiasis; (3) management with ERCP, biliodigestive diversion, sphincteroplasty, or laparoscopic transcystic or transductal approaches. Exclusion criteria were: (1) incomplete medical records; (2) absence of imaging or anatomical confirmation of common bile duct stones; and (3) biliodigestive diversion performed for other indications (e.g., cancer or iatrogenic post-cholecystectomy injury).

Abdominal ultrasound served as the first-line diagnostic modality. MRCP was performed selectively when further evaluation was required, while computed tomography (CT) contributed to the detection of some cases. Endoscopic ultrasound (EUS) was not available in this study, and ERCP was reserved primarily for therapeutic interventions.

**Figure 1:**
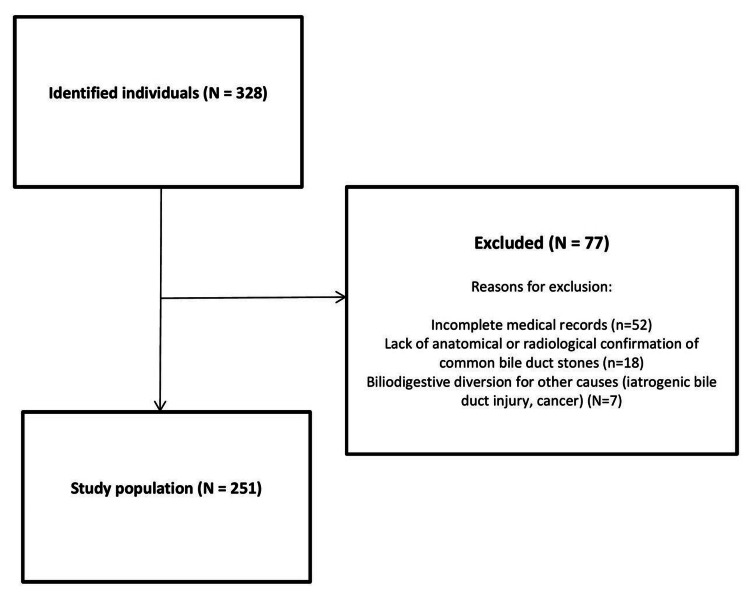
Flow diagram of the study population

A total of 328 participants were initially identified, of whom 77 were excluded. The main reasons for exclusion were incomplete medical records (n=52) and lack of anatomical or radiological confirmation of common bile duct stones (n=18). Thus, 251 participants were included in the present study. Figure 1 shows a flow diagram of the study population.

Data collection

Data were obtained from electronic medical records and, when necessary, complemented by review of physical charts. Laboratory parameters included complete blood count, liver enzymes, cholestatic markers, and coagulation profile, collected at admission according to institutional protocols. Procedural data were extracted from surgical and endoscopic reports.

Residual post-cholecystectomy choledocholithiasis was defined as the presence of main bile duct stones that were present in the common bile duct at the time of cholecystectomy but were not detected, typically presenting within two years after surgery. In contrast, primary post-cholecystectomy choledocholithiasis was defined as main bile duct stones that formed *de novo* in the bile duct, usually more than two years after the operation.

Complications were classified according to the Clavien-Dindo grading system [8], with events graded ≥III considered severe.

Treatment groups

Patients were stratified into three groups according to the therapeutic approach: (1) endoscopic (ERCP), (2) surgical (biliodigestive diversion, sphincteroplasty, or laparoscopic exploration), and (3) combined (ERCP + surgical). Outcomes included recurrence, complications, and the need for reintervention.

Upfront surgery was defined as the initial surgical approach for the treatment of common bile duct stones, with or without concomitant cholecystectomy, whereas surgery after ERCP referred exclusively to cases requiring operative intervention after failed ERCP or ERCP-related complications, not including situations where ERCP successfully treated choledocholithiasis and was followed only by cholecystectomy.

Statistical analysis

Descriptive statistics were expressed as frequencies for categorical variables and as measures of central tendency and dispersion for continuous variables (means and standard deviations or medians and interquartile range (IQR), when applicable). Categorical variables were compared using chi-square or Fisher’s exact test. Continuous variables were analyzed with Mann-Whitney or Kruskal-Wallis tests. Statistical significance was set at p<0.05. Analyses were performed using SAS System for Windows, version 9.2 (SAS Inc., Cary, NC, USA).

## Results

Sample characteristics

A total of 251 patients were included, with a predominance of female patients (61.8%, n=155). The overall median age was 56 years (IQR 39.8-71.0). Hypertension was the most prevalent comorbidity (29.1%), followed by type 2 diabetes (14.7%). Smoking and alcohol use were reported in 17.5% and 8.8% of patients, respectively. Previous cholecystectomy was documented in 31.9% (n=80), with a mean interval of 124.6 ± 131.5 months between surgery and clinical presentation of choledocholithiasis. Among these, 22.5% were classified as residual and 77.5% as primary choledocholithiasis. Nearly half of the patients (49.4%) were admitted on an urgent basis, while 47.8% were referred from outpatient care. The general demographic and clinical characteristics of the study population are summarized in Table 1.

**Table 1:** General characteristics of the study population N: number of individuals; ERCP: Endoscopic Retrograde Cholangiopancreatography; M: male; F: female; IQR: interquartile range

Variable	Exclusive ERCP (n=195, 77.8%)	Upfront surgery (n=28, 11.1%)	Post-ERCP surgery (n=28, 11.1%)	P-value
Age (years)	58 (IQR: 38-72)	52.5 (IQR: 43-69)	56 (IQR 48–65)	0.95
Sex	M 72 (36.9%) F 123 (63.1%)	M 10 (35.7%) F 18 (64.6%)	M 14 (50%) F 14 (50%)	0.39
Hypertension	54 (27.7%)	9 (32.1%)	10 (35.7%)	0.64
Diabetes	24 (12.3%)	5 (17.9%)	8 (28.6%)	0.07
Heart disease	15 (7.7%)	0	1 (3.6%)	0.34
Smoking	30 (15.4%)	3 (10.7%)	11 (39.3%)	0.24
Alcohol use	15 (7.7%)	3 (10.7%)	4 (14.3%)	0.66
Previous cholecystectomy	49 (25.1%)	18 (64.3%)	13 (46.4%)	0.0004
Post-cholecystectomy choledocholithiasis				
Residual	14 (28.6%)	2 (11.1%)	2 (15.4%)	0.25
Primary	35 (71.4%)	16 (88.9%)	11 (84.6%)	
Previous surgeries	90 (46.2%)	20 (71.4%)	20 (71.4%)	0.004
Origin				
Outpatient	92 (47.1%)	18 (64.3%)	17 (60.7%)	0.12
Emergency department	103 (52.9%)	10 (35.7%)	11 (39.3%)	

Previous cholangitis was significantly more frequent among surgical patients (46.4% in upfront surgery and 42.9% in surgery after ERCP) compared with the exclusive ERCP group (26.7%, p=0.04). There were no significant differences regarding history of pancreatitis (p=0.52).

Laboratory profile

Laboratory analysis at admission showed no significant differences between the groups regarding white blood cell count, hemoglobin, platelet count, creatinine, alkaline phosphatase, gamma-glutamyl transferase, or total bilirubin. However, international normalized ratio (INR) values were significantly lower in the upfront surgery group (p<0.001). In addition, aspartate aminotransferase (AST) and alanine aminotransferase (ALT) levels were significantly higher in the exclusive ERCP group compared with the surgical groups (p=0.003 and p=0.002, respectively). The full laboratory profile is presented in Table 2.

**Table 2:** Laboratory results at admission ERCP: Endoscopic Retrograde Cholangiopancreatography; INR: International Normalized Ratio; AST: Aspartate Aminotransferase; ALT: Alanine Aminotransferase; ALP: Alkaline Phosphatase; GGT: Gamma-Glutamyl Transferase.

Variable	Exclusive ERCP	Upfront surgery	Post-ERCP surgery	P-value
White blood cell count (×10^6^/µL)	9.3 ± 5.0	8.8 ± 2.9	7.2 ± 2.8	0.17
Hemoglobin (g/dL)	12.9 ± 2.2	13.5 ± 1.4	12.2 ± 1.5	0.20
Platelets (×10^6^/µL)	248.3 ± 110.5	272.3 ± 112.2	246.1 ± 83.2	0.55
Creatinine (mg/dL)	1.0 ± 1.1	0.8 ± 0.2	0.8 ± 0.3	0.31
INR	1.1 ± 0.3	0.8 ± 0.2	1.1 ± 0.1	<0.001
AST (U/L)	147.1 ± 142.4	55.1 ± 39.0	67.9 ± 51.1	0.003
ALT (U/L)	213.7 ± 222.9	78.6 ± 88.6	77.6 ± 58.9	0.002
ALP (U/L)	356.4 ± 481.1	574.5 ± 536.5	336.8 ± 268.7	0.22
GGT (U/L)	474.9 ± 428.5	555.6 ± 558.9	572.7 ± 569.0	0.59
Total bilirubin (mg/dL)	5.8 ± 7.1	4.1 ± 6.7	3.7 ± 4.6	0.34

Treatment distribution

Most patients (77.8%, n=195) underwent ERCP exclusively. Surgical treatment as the initial approach was performed in 11.1% (n=28), while an additional 11.1% (n=28) required surgery following failed ERCP. Overall, 56 patients underwent surgical management, most commonly due to ERCP failure (50%), previous surgery with duodenal exclusion (12.5%), or intrahepatic stones (8.9%). Other indications included large stones (7.1%), post-cholecystectomy strictures (3.6%), and miscellaneous causes (19.6%).

Surgical and endoscopic procedures

The most frequent surgical techniques were choledochoduodenostomy (44.6%) and Roux-en-Y hepaticojejunostomy (39.3%). Less frequent procedures included laparoscopic transcystic or transductal exploration and transduodenal sphincteroplasty. Regarding endoscopic management, a total of 223 ERCP procedures were analyzed. Sphincterotomy was the most common intervention (93.7%), followed by basket stone extraction (45.3%), biliary stent placement (35%), and infundibulotomy (8.5%).

Complications

Complications were observed in 24 patients (12.3%) after ERCP alone, nine patients (32.1%) after upfront surgery, and seven patients (25.0%) after surgery following ERCP. Morbidity was significantly higher in the upfront surgery and post-ERCP surgery groups (p=0.01). In the ERCP group, the most frequent complications were post-ERCP pancreatitis (n=9, 4.6%), cholecystitis (n=5, 2.6%), and cholangitis (n=4, 2.0%), followed by bleeding (n=3, 1.5%), duodenal perforation (n=2, 1.0%), pneumonia (n=1, 0.5%), and adynamic ileus (n=1, 0.5%). In the upfront surgery group, complications were mainly biliary fistula (n=3, 10.7%) and pneumonia (n=3, 10.7%), with additional cases of bleeding (n=2, 7.1%). Among patients undergoing surgery after ERCP, the most common events were biliary fistula (n=3, 10.7%) and pneumonia (n=2, 7.1%), followed by pancreatitis (n=2, 7.1%), cholangitis (n=1, 3.6%), cholecystitis (n=1, 3.6%), and bleeding (n=1, 3.6%).

Severe complications (Clavien-Dindo ≥III) occurred in 10 patients (5.1%) after ERCP, six patients (21.4%) after upfront surgery, and four patients (14.3%) after surgery following ERCP, with a significantly higher proportion in the surgical groups compared with ERCP (p<0.001). Mortality rates were similar across groups (5.1%, 7.1%, and 8.3%, p=0.85).

Length of stay

Hospital stay was significantly longer in surgical patients (9 ± 4.5 days in upfront surgery and 12 ± 12.3 days in surgery after ERCP) compared with exclusive ERCP (4.7 ± 5.7 days, p=0.0002). No significant differences were observed in ICU stay (p=0.46). The complete comparison is shown in Table 3.

**Table 3:** Patient history, stone-related characteristics, and in-hospital outcomes in choledocholithiasis ERCP: Endoscopic Retrograde Cholangiopancreatography; ICU: Intensive Care Unit; NA: Not applicable.

Variable	Exclusive ERCP	Upfront surgery	Post-ERCP surgery	P-value
Previous cholangitis	52 (26.7%)	13 (46.4%)	12 (42.9%)	0.04
Previous pancreatitis	38 (19.4%)	3 (10.7%)	5 (17.9%)	0.52
Number of prior ERCPs	1.3 ± 0.6	NA	1.7 ± 0.9	0.004
Size of largest stone (cm)	1.1 ± 0.8	1.9 ± 1.2	1.2 ± 0.7	<0.0001
Single vs. multiple stones	Single: 83 (42.6%) Multiple: 112 (57.4%)	Single: 6 (21.4%) Multiple: 22 (78.6%)	Single: 6 (21.4%) Multiple: 22 (78.6%)	0.02
Complications	24 (12.3%)	9 (32.1%)	7 (25.0%)	0.01
In-hospital mortality	10 (5.1%)	2 (7.1%)	2 (8.3%)	0.85
Length of stay (days)	4.7 ± 5.7	9.0 ± 4.5	12.0 ± 12.3	0.0002
ICU stay (days)	0.6 ± 3.6	1.7 ± 2.3	0.8 ± 1.4	0.46

## Discussion

In this comparative analysis of therapeutic groups, patients who required surgical treatment, either as upfront surgery or after ERCP, had higher rates of previous cholecystectomy and prior abdominal surgeries compared with those managed exclusively endoscopically. These findings suggest that surgical history may be associated with greater anatomic complexity, scarring, or biliary distortion, factors that reduce the success of endoscopic access and increase the likelihood of surgical intervention. Previous studies have similarly reported that surgical history, particularly cholecystectomy, is a risk factor for residual or recurrent choledocholithiasis and directly influences therapeutic strategy [2,9-13].

Regarding laboratory findings, INR values were significantly higher in the exclusive ERCP and ERCP plus surgery groups. This may reflect impaired hepatic function or altered coagulation factor synthesis secondary to prolonged biliary obstruction. Although elevated INR alone is not a contraindication for endoscopic therapy, it is associated with a higher risk of post-sphincterotomy bleeding, underscoring the importance of pre-procedure coagulation assessment [14,15].

Liver enzyme levels (AST and ALT) were also significantly higher in the ERCP group, likely reflecting greater hepatocellular injury due to acute or intermittent biliary obstruction or prior cholangitis. The literature supports that marked transaminase elevation in choledocholithiasis often indicates a sudden obstruction by an impacted stone, frequently leading to emergency presentation, consistent with the higher rate of urgent admissions observed in this group [16-18].

Patients requiring surgery after failed ERCP underwent a greater number of endoscopic attempts compared with those managed successfully by ERCP alone, highlighting the technical challenges of refractory cases. International series have described similar patterns, with failure of endoscopic therapy often related to large, multiple, or intrahepatic stones [19]. Indeed, in our cohort, stone size was greater in upfront surgical cases, whereas multiple stones were more common among surgically treated patients overall. Both factors are well-recognized determinants of ERCP failure and justification for surgical management [14-16].

Acute cholangitis prior to intervention was also more frequent among surgical patients, reflecting greater clinical severity. The presence of cholangitis increases perioperative risk and usually requires a more definitive resolution of the obstruction, explaining the preference for surgical approaches in such cases [19].

Post-procedural complications were significantly higher in surgical groups. This can be interpreted both as a reflection of greater clinical and anatomical complexity and as an inherent feature of surgical interventions, which carry higher morbidity compared with ERCP. Nevertheless, in-hospital mortality did not differ significantly among groups, suggesting that appropriate therapeutic selection ensures favorable survival outcomes even in severe and complex cases [20-22].

Length of hospital stay was significantly longer in surgical patients, both for upfront and post-ERCP surgery. This is consistent with prior reports and reflects not only greater disease severity but also the impact of surgery itself, which requires longer recovery and is associated with higher complication rates. By contrast, ICU stay did not differ significantly, suggesting that while overall morbidity and hospitalization length may vary, the need for intensive care support is not substantially influenced by the treatment modality [21-23]. Nonetheless, the interpretation of differences in complications and length of stay must be made with caution, as patient allocation was not randomized. Although upfront surgery cases may appear to represent technically easier patients, this is not necessarily the case, as selection can be influenced by local expertise, patient comorbidities, and resource availability. Conversely, surgery after failed ERCP often reflects more complex clinical scenarios, such as difficult anatomy, large or impacted stones, or procedure-related complications, which may partly explain the higher observed morbidity in this group.

Taken together, our findings reinforce ERCP as the first-line treatment for most patients with choledocholithiasis, offering high success rates with lower morbidity and shorter hospitalization. However, the presence of large or multiple stones, prior surgical history, and severe cholangitis frequently shifts management toward surgical approaches. Despite their association with higher complication rates and prolonged hospital stays, surgery remains essential for achieving definitive resolution in complex cases [24,25].

According to evidence provided by population-level and single-center studies, baseline ERCP-related mortality for uncomplicated choledocholithiasis is low (~0.3-0.5%) [26]. However, mortality rises with greater physiologic burden and delayed biliary drainage in acute cholangitis, with early ERCP consistently associated with lower in-hospital and 30-day mortality [27,28]. In public healthcare tertiary cohorts from Brazil, meaningful complication rates and occasional fatalities after ERCP have been reported [29]. Moreover, a cross-sectional study from a tertiary university hospital in Brazil demonstrated that acute cholangitis was frequently underdiagnosed: while only 9.9% of cases had a clinical diagnosis recorded, and application of the Tokyo Guidelines revealed a real prevalence of approximately 43%, underscoring the deficiencies in referral pathways and diagnostic accuracy within the public healthcare system [30]. When endoscopic clearance fails and surgical rescue is required, short-term mortality of around 3% has been observed, although highly specialized centers can report near-zero operative mortality with careful selection [1,31]. Our relatively high mortality reflects the complex case mix of a tertiary referral center, including severe cholangitis and patients requiring salvage surgery after ERCP failure. Promising approaches, such as laparo-endoscopic rendezvous techniques, have been reported internationally with promising outcomes, although their availability in the Brazilian public system remains limited.

This study is limited by its retrospective, single-center design, reliance on medical records, and the use of a convenience sample. A convenience sample may introduce selection bias, as patients included reflect those who presented to our institution and met the inclusion criteria, rather than a systematically recruited cohort. As a result, the findings may not be representative of broader populations or different practice settings, thereby limiting generalizability. Data collection relied on medical records, which may be subject to missing or incomplete information. The study population reflects the experience of a tertiary referral hospital, which may limit generalizability to other settings with different patient profiles and resource availability. In addition, treatment allocation was not randomized but determined by clinical presentation and institutional resources, which may have introduced selection bias. Within the constraints of sample size and study design, the extended study period still offers insight into real-world therapeutic strategies for choledocholithiasis.

## Conclusions

This retrospective analysis of patients with choledocholithiasis treated at a tertiary public university hospital indicates that ERCP was commonly employed as the primary therapeutic modality, while surgery was required in a minority of cases, often in the setting of greater clinical and anatomical complexity. Surgical management appeared to involve longer hospital stays and higher complication rates, though mortality did not differ significantly. These observations should be regarded as descriptive of this institutional experience rather than broadly generalizable, and they emphasize the importance of recognizing potential predictors of endoscopic failure and of establishing clear referral pathways within public health systems.
